# Evaluation of the Rapid and Cartridge-Based STANDARD™ M10 STI Panel: Analytical and Clinical Performance for Multiplex STI Detection

**DOI:** 10.3390/microorganisms14030631

**Published:** 2026-03-11

**Authors:** Massimiliano Guerra, Martina Brandolini, Laura Dionisi, Claudia Colosimo, Giulia Gatti, Alessandra Mistral De Pascali, Endah Indriastuti, Ludovica Ingletto, Anna Marzucco, Maria Sofia Montanari, Laura Grumiro, Giorgio Dirani, Silvia Zannoli, Alessandra Scagliarini, Monica Cricca, Vittorio Sambri

**Affiliations:** 1 Unit of Microbiology, The Greater Romagna Area Hub Laboratory, 47522 Cesena, Italy; anna.marzucco@auslromagna.it (A.M.); giorgio.dirani@auslromagna.it (G.D.); silvia.zannoli@auslromagna.it (S.Z.); monica.cricca3@unibo.it (M.C.); vittorio.sambri@unibo.it (V.S.); 2 Department of Medical and Surgical Sciences (DIMEC), University of Bologna, 40138 Bologna, Italy; martina.brandolini3@unibo.it (M.B.); laura.dionisi2@unibo.it (L.D.); claudia.colosimo2@unibo.it (C.C.); giulia.gatti12@unibo.it (G.G.); alessandra.depascal3@unibo.it (A.M.D.P.); ludovica.ingletto@unibo.it (L.I.); mariasofia.montanar2@unibo.it (M.S.M.); laura.grumiro2@unibo.it (L.G.); alessand.scagliarini@unibo.it (A.S.); 3 Department of Medicine, Faculty of Medicine and Health, Institut Teknologi Sepuluh Nopember, Surabaya 60111, Indonesia; endah.indriastuti@its.ac.id

**Keywords:** STI, multiplex PCR, cartridge-based diagnostics, point-of-care testing, STANDARD™ M10, molecular diagnostics, analytical performance, clinical evaluation

## Abstract

Sexually transmitted infections (STIs) remain a major global public health concern, with more than one million new cases acquired daily and increasing antimicrobial resistance compromising effective control strategies. Rapid, accurate and multiplex molecular diagnostics are therefore essential to support timely clinical management and public health surveillance. This study evaluated the analytical and clinical performance of the cartridge-based STANDARD™ M10 STI Panel (SD Biosensor, Republic of Korea), a fully automated, random-access real-time PCR assay capable of detecting eight STI-related pathogens within approximately 64 min. A total of 150 residual clinical specimens were retrospectively analysed, including vaginal, rectal, urethral and oropharyngeal swabs, as well as seminal fluids, and results were compared with those obtained using the Allplex™ STI Essential Assay (Seegene, Republic of Korea), which shares six common targets. The STANDARD™ M10 STI Panel demonstrated high diagnostic accuracy, with sensitivity ranging from 89.5% to 100%, specificity from 98.2% to 100% and overall accuracy between 98% and 100%. Agreement between the two assays was almost perfect, with Cohen’s κ values ranging from 0.91 to 1.00. Analytical sensitivity was further confirmed through verification of the limits of detection using quantified reference standards. Although validated for urine samples, the assay also showed robust performance on alternative clinical matrices, particularly vaginal swabs. Overall, these findings indicate that the STANDARD™ M10 STI Panel represents a reliable and practical tool for STI diagnosis, combining rapid turnaround time, minimal hands-on requirements and broad pathogen coverage in both centralized and near-patient testing settings.

## 1. Introduction

The global epidemics of HIV, viral hepatitis and sexually transmitted infections (STIs) continue to constitute a major burden on public health worldwide, accounting collectively for approximately 2.5 million deaths annually, as reported by the World Health Organization (WHO) [1].

In particular, STIs are caused by more than 30 distinct pathogens, including bacteria, viruses, parasites and are mainly transmitted through sexual contact, including vaginal, anal and oral intercourse [2].

More than one million STIs are acquired every day [3]. In 2020, it is estimated that 374 million new infections occurred with one of four bacterial or parasitic STIs that can be cured with available antimicrobials.

In addition, rising trends in antimicrobial resistance have been observed for *Neisseria gonorrhoeae* and *Mycoplasma genitalium* [1]. Overall, STIs are associated with numerous adverse health outcomes, including infertility, pelvic inflammatory disease (PID), chronic pelvic pain and increased risk of certain cancers [4,5]. Several STIs can negatively affect pregnancy outcomes, increasing the risk of spontaneous abortion, preterm labor, stillbirth and vertical transmission to the newborn, either in utero or during delivery [6,7]. The psychosocial and economic burden of STIs—often underestimated—can significantly reduce individual quality of life [3].

The World Health Organization launched a new Global Strategy on STIs for 2022–2030, placing renewed emphasis on research, innovation and evidence-based policies as pillars of the international response. In 2022, the WHO initiated a global research priority-setting exercise to identify the most urgent areas of investigation to address the public health burden of STIs. The initiative pursued three main objectives: (1) to define global STI research priorities through inclusive consultations with experts and stakeholders across all WHO regions; (2) to provide guidance for adapting these global priorities to regional and local contexts; (3) to disseminate the priorities widely, offering strategic direction for STI research through 2030. Research aligned with these priorities is expected to give a significant contribution toward achieving the 2030 Sustainable Development Goals (SDGs), particularly those aimed at universal access to sexual and reproductive healthcare and the control of communicable diseases, ultimately promoting health and well-being for all [8].

Diagnostic approaches exhibit substantial differences between high-income and low- and middle-income countries (HICs and LMICs). In high-income settings, accurate laboratory-based testing—particularly for asymptomatic infections—is standard practice [9].

Conversely, LMICs rely on syndromic management, a clinical approach based on the presence of symptoms rather than laboratory confirmation [3]. While cost-effective and easy to implement, syndromic management lacks sensitivity, as many STIs are asymptomatic [10].

Diagnostic approaches for STIs primarily rely on direct pathogen detection methods, such as nucleic acid amplification tests (NAATs), which represent the current gold standard. Although host immune response or metabolomic-based assays have been explored in research settings, they are not routinely implemented in clinical STI diagnostics [11]. However, complex clinical samples may contain inhibitory substances that interfere with detection accuracy. Newer generations of commercial molecular assays have progressively improved nucleic acid purification and internal inhibition control mechanisms, thereby reducing the impact of such inhibitors on diagnostic accuracy [12]. Nucleic acid amplification tests (NAATs) are considered the gold standard for STI diagnosis, offering high sensitivity and specificity even in asymptomatic infections. According to WHO recommendations, first-void urine in men and vaginal swabs in women are the preferred specimen types for molecular detection of *Chlamydia trachomatis*, *Neisseria gonorrhoeae* and *Mycoplasma genitalium* [11].

High-prevalence STI pathogens—including *Chlamydia trachomatis* (CT), *Neisseria gonorrhoeae* (NG), *Trichomonas vaginalis* (TV), *Ureaplasma urealyticum* (UU), *Mycoplasma hominis* (MH) and *Mycoplasma genitalium* (MG)—present considerable diagnostic challenges due to frequent co-infection and nonspecific or absent symptoms [13,14,15,16,17,18,19]. Therefore, multiplex molecular assays capable of detecting multiple STI pathogens simultaneously are highly desirable for accurate and timely diagnosis [20].

In this context, the present study was conducted at the Microbiology Unit of the Greater Romagna Laboratory Hub in Pievesestina (Cesena, Emilia-Romagna, Italy). The study aimed to evaluate the diagnostic performance of the STANDARD™ M10 STI Panel (SD Biosensor Inc., Suwon, Republic of Korea), a molecular assay designed for the simultaneous detection and differentiation of multiple sexually transmitted infection pathogens. Specifically, the panel targets *Chlamydia trachomatis* (CT), *Neisseria gonorrhoeae* (NG), Herpes simplex virus type 1 (HSV-1), Herpes simplex virus type 2 (HSV-2), *Mycoplasma genitalium* (MG), *Mycoplasma hominis* (MH), *Trichomonas vaginalis* (TV), and *Ureaplasma urealyticum* (UU). The STANDARD™ M10 platform is a fully automated, cartridge-based molecular system designed for both centralized laboratories and decentralized or point-of-care (POC) testing. Its self-contained workflow and minimal hands-on time enable rapid and reliable STI detection, making it suitable for use in clinical laboratories as well as near-patient settings.

The STANDARD™ M10 STI Panel is intended to be used on urine samples; in this study, however, the performance of the kit will mainly be evaluated on vaginal swabs and other common matrices for the detection of STI-related pathogens. Although the STANDARD™ M10 STI Panel is intended to be used on urine samples, several studies have demonstrated the feasibility of molecular detection of sexually transmitted pathogens using alternative specimen types, including vaginal, rectal, urethral and oropharyngeal swabs [21,22,23].

This study was carried out in two sub-studies. In the first one, the diagnostic performance of the STANDARD™ M10 STI Panel was compared with the Allplex™ STI Essential Assay (Seegene, Seoul, Republic of Korea) using a dataset of 150 clinical samples.

In the second sub-study, the Limit of Detection (LoD) of the STANDARD™ M10 STI Panel was evaluated for each target organism detected by the kit using a set of quantified commercial standards.

## 2. Materials and Methods

### 2.1. Study Design, Population and Sample Collection

This retrospective observational study analysed 150 residual, anonymized clinical samples collected between February and May 2025 from individuals presenting with symptoms suggestive of a sexually transmitted infection (STI) or seeking medical evaluation following unprotected sexual intercourse. Samples were obtained from diagnostic laboratories located in the Romagna area (provinces of Forlì-Cesena, Rimini and Ravenna, north-eastern Italy) and referred to the Unit of Microbiology at the Greater Romagna Area Hub Laboratory (Cesena, Italy) for routine molecular STI screening using the Allplex™ STI Essential Assay. Detailed demographic data and comprehensive clinical information, including symptom severity and duration, were not consistently available for all subjects and were therefore not used for stratified analyses. This limitation reflects the retrospective nature of the study and the use of residual samples collected during routine diagnostic care. Following completion of routine diagnostics, the residual clinical material from each specimen was used for comparative testing with the STANDARD™ M10 STI Panel. Although testing was performed retrospectively, both assays were conducted on the same biological matrix, ensuring analytical comparability. Samples were included consecutively according to availability in the diagnostic workflow, including both positive and negative cases, and were selected solely based on the presence of sufficient residual volume and adequate sample integrity for retesting. To minimize potential sources of bias, the study design relied on the consecutive inclusion of routine clinical specimens rather than preselected positives. All comparative analyses were performed in a blinded manner with respect to the reference method, and every effort was made to ensure proper storage and transport conditions (2–8 °C) to preserve nucleic acid stability and prevent degradation. This approach was intended to maintain analytical consistency between the two assays and to reflect real-world laboratory conditions as closely as possible.

Inclusion criteria comprised biological specimens that tested positive for at least one of the following pathogens during routine diagnostic procedures: *Chlamydia trachomatis* (CT), *Neisseria gonorrhoeae* (NG), *Mycoplasma genitalium* (MG), *Mycoplasma hominis* (MH), *Trichomonas vaginalis* (TV), or *Ureaplasma urealyticum* (UU). Samples that tested negative for all included targets were also enrolled as negative clinical specimens to serve as comparators in performance evaluation. All samples were fully anonymized prior to inclusion, in compliance with the ethical standards of the Romagna Local Health Authority and the Italian Code of Conduct for the Use of Health Data for Scientific Research (Veneto Region, ULSS 9 Scaligera). Because this was a retrospective study using archived, anonymized specimens, no specific ethical approval or informed consent was required, consistent with local and national regulations (protocol code AVR-PPC P09, rev.2; Burnett et al., 2007) [24].

**Figure 1:**
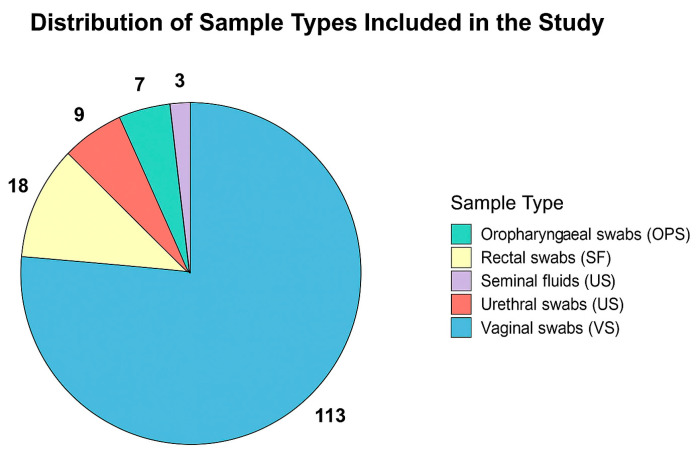
Distribution of samples types included in the study.

A total of 113 vaginal swabs (VSs), 18 rectal swabs (RSs), 9 urethral swabs (USs), 7 oropharyngeal swabs (OPSs) and 3 seminal fluids (SFs) were analysed in this study (the distribution of samples is detailed in Figure 1). All swabs were collected using FLOQSwabs (Copan, Brescia, Italy). Each specimen was placed immediately after collection into a universal transport medium (UTM™, Copan, Brescia, Italy) to preserve nucleic acid integrity. Samples were transported to the central laboratory under controlled refrigerated conditions (2–8 °C) and processed within 24 h of receipt. When immediate processing was not feasible, specimens were temporarily stored at +4 °C to minimize nucleic acid degradation.

### 2.2. Allplex™ STI Essential Assay-Index Test

Allplex™ STI Essential Assay (Seegene, Seoul, Republic of Korea) is a batch-based molecular test that enables the simultaneous processing of up to 94 samples. When run at full capacity, the total turnaround time is approximately 4 h and 40 min, comprising both nucleic acid extraction and real-time PCR amplification. The extraction and PCR setup are carried out using the STARlet IVD automated platform (Hamilton Company, Bonaduz, Switzerland) and require around 2 h and 40 min, with timing dependent on the number of samples loaded. PCR amplification has a fixed runtime of 2 h, regardless of sample volume.

The assay targets eight sexually transmitted infection pathogens, namely *Chlamydia trachomatis* (CT), *Neisseria gonorrhoeae* (NG), *Mycoplasma genitalium* (MG), *Mycoplasma hominis* (MH), *Trichomonas vaginalis* (TV), *Ureaplasma urealyticum* (UU), Herpes simplex virus type 1 (HSV-1) and Herpes simplex virus type 2 (HSV-2). Detection relies on amplification of pathogen-specific genetic regions, including *ompA* for *Chlamydia trachomatis*, *porA* for *Neisseria gonorrhoeae*, *MgPa* for *Mycoplasma genitalium*, *YidC* for *Mycoplasma hominis*, β-tubulin for *Trichomonas vaginalis*, the multiple banded antigen (MBA) gene for *Ureaplasma urealyticum*, and glycoprotein I and glycoprotein B for Herpes simplex virus type 1 and Herpes simplex virus type 2, respectively, as declared by the manufacturer. The assay can be performed on various specimen types including urine, vaginal swabs, oropharyngeal swabs, rectal swabs and seminal fluid.

For each sample, 300 µL of clinical material was processed and nucleic acids were extracted using the STARMag 96 X 4 Universal Cartridge Kit (Seegene, Seoul, Republic of Korea), with final elution in 100 µL. PCR amplification and detection were performed according to the manufacturer’s instructions using the STARlet platform and the CFX96 real-time PCR instrument (Bio-Rad, Feldkirchen, Germany). For each positive sample, the corresponding cycle threshold (Ct) value was reported [25].

### 2.3. STANDARD™ M10 STI Panel

The assay is based on nucleic acid amplification technology using real-time PCR and is performed within a single-use, self-contained cartridge that integrates all necessary reagents, including lysis buffers, nucleic acid purification components and amplification mix. Each STANDARD™ M10 STI Panel cartridge is designed for qualitative in vitro diagnosis, ensuring a closed, contamination-free environment for the automated processing of clinical specimens. Each cartridge allows the processing of a single sample and a single test last about 64 min. In case of positive result, the test provides the cycle threshold (ct) for each target identified.

The biological matrix for which the kit is validated is urine. However, in this study we tested the kit on other biological materials commonly used for the detection of pathogens identified by the kit: VS, US, RS, OPS and SF. The materials used are listed in the paragraph entitled Study Design, Population and Sample Collection.

The STANDARD™ M10 STI Panel kit can be carried out exclusively on STANDARD™ M10 instruments. The system consists of a console (STANDARD™ M10 Console) and a variable number of independent modules (STANDARD™ M10 modules), ranging from 1 to 8. An instrument configured with eight modules was used for the experiments in this study. The STANDARD™ M10 system is a fully automated, on-demand molecular platform that enables continuous sample loading, random-access testing, and automated nucleic acid extraction, amplification and detection within a closed cartridge-based workflow.

### 2.4. Limit of Detection (LoD) Verification

The Limit of Detection (LoD) for each target pathogen was verified using the quantified commercial reference standards and the corresponding manufacturer-declared LoD values summarized in Table 1. For each organism, Table 1 reports (i) the manufacturer-declared LoD concentration, (ii) the nominal concentration of the quantified reference material provided by the supplier, and (iii) the dilution series tested in this study. Each standard was serially diluted in 0.9% sodium chloride solution (B. Braun, Melsungen, Germany) to obtain five testing levels—4×, 2×, 1×, 0.5×, and 0.25× of the manufacturer-declared LoD—which were tested in triplicate across three independent experiments. The experimental results were compared with the manufacturer-declared LoD values reported in Table 1 to verify the analytical sensitivity for each target organism [26].

Analytical verification was performed using commercially available quantified reference materials and DNA controls. *Chlamydia trachomatis* and *Neisseria gonorrhoeae* were assessed using the AccuTrak™ CT/NG Verification Panel (SeraCare Life Sciences Inc., Milford, MA, USA), while *Trichomonas vaginalis* and *Mycoplasma genitalium* were evaluated using the AccuTrak™ TV/MG Verification Panel (SeraCare Life Sciences Inc., Milford, MA, USA). Herpes simplex virus type 1 and Herpes simplex virus type 2 were verified using NATtrol™ Herpes Simplex Virus Type 1 and Type 2 controls (ZeptoMetrix Corporation, Buffalo, NY, USA). *Mycoplasma hominis* and *Ureaplasma urealyticum* were assessed using Amplirun^®^ DNA controls (Vircell Microbiologists, Granada, Spain).

### 2.5. Data Analysis

Upon completion of the thermal cycling protocol, the real-time PCR software (STANDARD™ M10 Console, software version V001.011.001) automatically calculates the cycle threshold (Ct) values for each sample. Samples exhibiting amplification curves that crossed the fluorescence threshold within the assay-specific Ct range were automatically classified as positive, whereas samples with no amplification within this range were considered negative. Negative results were interpreted as either true negatives or indicative of pathogen loads below the assay’s limit of detection. Ct threshold values and acceptance criteria were established in accordance with internal and positive controls specific to each assay run. Final test results were automatically generated and interpreted by the STANDARD™ M10 Console and visually presented on the “Review screen”.

### 2.6. Statistical Analysis

Statistical analyses were conducted using Microsoft Excel (version 16.0, Microsoft Corporation, Redmond, WA, USA, 2021). For each sample, results obtained with the STANDARD™ M10 STI Panel were compared to those generated by the Seegene Allplex™ STI Essential Assay, which served as the reference standard. Key diagnostic performance metrics—including sensitivity, specificity, positive predictive value (PPV), negative predictive value (NPV), overall accuracy, and Cohen’s κ coefficient—were calculated individually for each targeted pathogen. Sensitivity and specificity were determined from 2 × 2 contingency tables, and numerators and denominators (*n*/*N*) indicate the number of concordant positive or negative results relative to the total number of expected positive or negative samples. Two-tailed 95% confidence intervals (CIs) were calculated for each proportion using the Wilson score method, while 95% CIs for Cohen’s κ were estimated using the asymptotic (Fleiss) approach. Cohen’s κ coefficient was interpreted according to Landis and Koch’s criteria to evaluate the level of agreement between methods beyond chance. Samples that remained discordant after retesting were retained in the study database and included in the statistical analyses. The comprehensive results of these analyses are detailed in the subsequent sections.

## 3. Results

### 3.1. Evaluation of STANDARD™ M10 STI Panel Using Clinical Samples

In an initial sub-study, 150 samples were analyzed. These included 113 vaginal swabs, 18 rectal swabs, 9 urethral swabs, 7 oropharyngeal swabs, and 3 seminal fluid samples. The distribution of sample types is illustrated in Figure 1, and the corresponding absolute counts and number of positive samples detected by either method are summarized in Table 2.

Of these, 50 samples identified negative by the routine test were confirmed by STANDARD™ M10 STI panel kit including HSV-1 and HSV-2, which are identified exclusively by the SD Biosensor’s test (Table S1 Supplementary Material).

The remaining 100 samples were positive for at least one target among CT-NG-TV-MG-MH-UU, which are the common targets between Allplex™ STI Essential Assay and STANDARD™ M10 STI Panel.

Of these 100 samples, 23 were positive for *Ureaplasma parvum* (UP), a target included only in the Allplex™ STI Essential Assay and not in the STANDARD™ M10 STI Panel. This information was reported for completeness and to confirm that the presence of *Ureaplasma parvum* did not interfere with the performance of the investigational assay, as no cross-reactivity was observed. The Ct values for *Ureaplasma parvum* detection ranged from 18.51 to 39.90, and none of these samples showed discrepant results between the two methods. The complete list of samples and respective ct values for the UP target are reported in Table 3.

Among the 100 positive samples, without considering discrepant ones, 22 were identified by simultaneous positivity to two targets and 5 to three targets. (Table S2 Supplementary Material). Aggregating the data in Tables S2 and S3, it was possible to calculate the sensitivity, specificity, NPV, PPV, accuracy and Cohen’s kappa (κ) values that are present in Table 4. These results allowed to calculate sensitivity and specificity; these can vary depending on the organism identified between 89.47–100% and 98.23–100%, respectively. Similarly, the values of NPV and PPV are in the ranges of 92.86–100% and 94.44–100%, respectively. Accuracy values are included in the range 98–100%, indicating almost a total overlapping of the two tests in terms of results (Table 4). It should be noted that the PPVs and NPVs reported here reflect the internal composition of the study dataset and are not intended to represent population-level predictive performance. As these parameters are inherently dependent on disease prevalence, they were calculated only as descriptive analytical indicators to complement sensitivity and specificity, which remain the primary metrics for comparative evaluation between assays. Finally, Cohen’s kappa analysis comparing the performances of the two tests determined a **κ** value for each target organism included in the range 0.91–1, which indicates a rate of “almost perfect” [27].

Out of a total of 150 clinical samples, 9 (6%) presented discrepancies between the two diagnostic methods. Notably, two of these samples exhibited discrepancies for two targets simultaneously. In total, there were four samples in which the STANDARD™ M10 STI Panel failed to detect five target organisms that were instead identified by the Allplex™ STI Essential Assay. Conversely, the STANDARD™ M10 STI Panel identified six target organisms in five samples that were not detected by the Allplex™ assay. The discrepancies observed are likely attributable to low bacterial load, as suggested by the high cycle threshold (Ct) values (>30) recorded for most of the discordant targets (Table 5). Only two samples—specifically, samples 104 and 149—showed Ct values <30, indicative of higher bacterial loads. An additional factor potentially contributing to these discrepancies may be the type of biological matrix, as most discordant samples (4 out of 9) originated from rectal swabs (RS), as reported in Table 5.

### 3.2. Evaluation of STANDARD™ M10 STI Panel Using Quantified Standards

In the second sub-study, we analyzed the ability of the STANDARD™ M10 STI panel test to identify known quantified standards of the test target organisms at concentrations around that of LoD. Specifically, the lowest (0.5× and 0.25× LoD) and highest (4× and 2× LoD) concentrations were analyzed in addition to the LoD concentration by executing 3 independent triplicates. The test identified all 4× and 2× concentrations except for one CT replicate (Table 6). LoD concentrations of HSV-1 and HSV-2 were identified in all three independent replications. The LoD concentration of CT, TV, MH, UU was only identified in one replication, while for MG and NG targets were identified in 2 out of 3 replications. Concentrations below the LoD were not identified apart from a replication of the 0.5 LoD concentration for MG and HSV-1 (Table 6). The Ct trend showed the expected inverse correlation between Ct values and target concentrations, with Ct values increasing as target concentrations decreased.

## 4. Discussion

According to the European Centre for Disease Prevention and Control (ECDC), cases of sexually transmitted infections (STIs) continue to rise across Europe [28], highlighting the need for accurate diagnostic tools to support infection control and public health surveillance. In this context, accurate diagnosis of STI-related pathogens is essential for infection control and public health surveillance [17]. Against this background, the present study evaluated the diagnostic performance of the STANDARD™ M10 STI Panel under routine laboratory conditions, focusing in particular on the use of clinical matrices other than urine, which is the only validated sample type included in the manufacturer’s instructions. The use of non-urine matrices in this study should therefore be considered exploratory and intended to generate preliminary performance data under real-world laboratory conditions.

The inclusion of HSV-1 and HSV-2 among the targets reflects the configuration of current CE-IVD syndromic STI panels. However, clinical interpretation of HSV results must always be contextualized to the anatomical site and clinical presentation, as lesion-derived samples remain the standard for HSV diagnosis.

The Allplex™ STI Essential Assay was selected as the comparator as it represents a well-established laboratory reference method for multiplex STI molecular diagnostics. Although no currently available commercial assay covers the exact same panel of targets included in the STANDARD™ M10 STI Panel, the Allplex™ assay shares six of the eight pathogens investigated in this study (CT, NG, TV, MG, MH and UU), making it the most suitable comparator among CE-IVD–marked molecular assays currently in use [29]. In addition, the Allplex™ STI Essential Assay has been widely adopted in routine diagnostic laboratories and has been evaluated in several clinical performance studies, demonstrating high analytical sensitivity, specificity and robustness across multiple specimen types [30].

Other commercially available multiplex STI assays, including the Vircell Sexually Transmitted Disease PCR panel (Vircell Microbiologists, Granada, Spain), the LabTurbo AIO Sexually Transmitted Diseases Panel (Taigen Bioscience Corporation, Taipei, Taiwan), the VIASURE Sexually Transmitted Diseases Real-Time PCR Detection Kit (CerTest Biotec, Zaragoza, Spain), and the RayBio^®^ Sexually Transmitted Disease (STD) Multiplex PCR Kit (RayBiotech, Norcross, GA, USA), differ substantially in assay design, target composition, workflow complexity and level of automation [31,32]. In particular, many of these platforms require batch-based processing, increased hands-on time, or lack random-access capability, which limits their suitability as direct comparators for an on-demand, cartridge-based system such as the STANDARD™ M10 platform.

Analysis of performance revealed an almost perfect concordance between the two assays, regardless of the biological matrix tested. (Table 3, Table 4, Tables S1 and S2). Cohen’s kappa values indicate a concordance rate of “almost perfect” and accuracy is included in the range 98–100% (Table 4). Mainly, the performance of the kit was assessed using vaginal swabs (113 out of 150 samples); then we enriched our dataset with rectal swabs (18/150), urethral swabs (9/150), oropharyngeal swabs (7/150) and seminal fluids (3/150—see Figure 1). Notably, there were 9 discrepant results and only two of these were obtained by analysing VS; the percentage of discrepant results obtained by using VS was 1.76%. These findings suggest that the biological matrix may influence assay performance, particularly for specimen types characterized by increased analytical complexity. Discrepant results between the two assays are likely attributable to a combination of analytical and pre-analytical factors rather than to systematic assay failure [33]. Several discrepant samples showed high Ct values, consistent with low target concentrations close to the analytical limit of detection, a condition known to increase stochastic variability and reduce inter-assay concordance. In addition, complex specimen matrices such as rectal and oropharyngeal swabs may contain PCR inhibitors or heterogeneous microbial backgrounds that can negatively impact amplification efficiency [34]. Finally, minor differences in assay design and genomic target selection may contribute to discordant detection in low-titer samples [35].

However, while the number of vaginal swabs analysed was substantial, the same cannot be said for the other biological matrices. A limitation of this study is the relatively small number of rectal, urethral, oropharyngeal swabs and seminal fluid samples included (Figure 1). The decision to include biological matrices other than vaginal swabs was aligned with the objective of assessing the performance of the STANDARD™ M10 STI Panel under routine clinical conditions. More comprehensive studies are planned to further evaluate assay performance across these specimen types. Accordingly, the present findings should be interpreted as exploratory for certain matrices and targets, particularly where the number of positive samples was limited.

**Figure 2:**
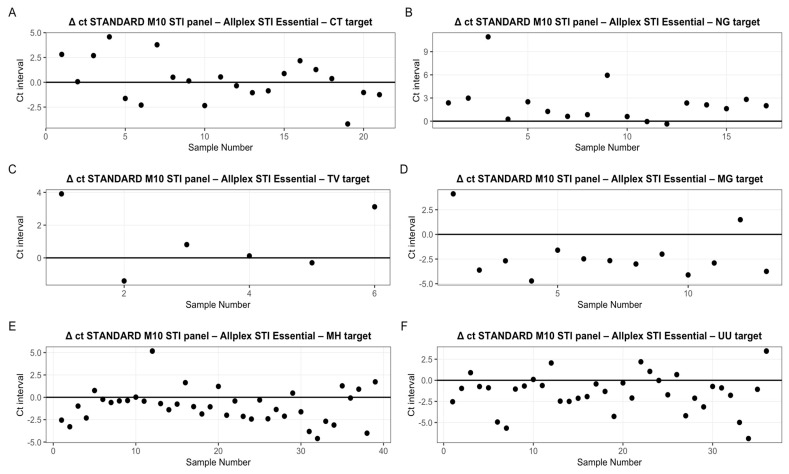
Analysis of the ct obtained for each identified target concordant between the two kit under investigation. The ΔCt were obtained by subtracting the values identified by the STANDARD™ M10 STI panel from those indicated by Seegene. In general, it is observed that the STANDARD™ M10 STI panel identifies MG, MH and UU at lower ct (**D**–**F**). The opposite trend is observed for NG (**B**). In contrast, there are no significant differences for the targets CT, TV (**A**,**C**).

Based on data reported in Table S1, Ct values were further explored to describe relative detection trends between the two assays, excluding discrepant results. In particular, ΔCt values were calculated for each identified target by subtracting the Ct values obtained with the STANDARD™ M10 STI Panel from those obtained with the Allplex™ STI Essential Assay. As shown in Figure 2, the STANDARD™ M10 STI Panel detected MG, MH and UU at lower Ct values compared to the comparator assay, suggesting higher analytical sensitivity for these targets. In contrast, the opposite trend was observed for NG, while CT and TV showed largely overlapping Ct distributions between the two platforms. Although Ct values may differ across molecular platforms due to differences in amplification chemistry and fluorescence detection algorithms, this analysis was intended to provide a descriptive comparison of detection patterns rather than a quantitative assessment of assay equivalence [34]. Accordingly, Ct values were not used for inferential statistical testing.

Regarding the presence of UP in the sample, our data show that in 23 cases, it, regardless of the bacterial load (see ct value in Table 3), did not interfere with the performance of the STANDARD™ M10 STI panel kit (Table 3). Likewise, the presence of HSV-1 and HSV-2 detected in 5 samples already positive for other pathogens did not interfere with the final result (Table S3 Supplementary Material). No evidence of analytical cross-reactivity or target suppression was observed in either assay, and high microbial loads did not result in false-negative results or impaired target detection. Intriguingly in sample 120 STANDARD™ M10 STI panel test not only identified MH which was missed by the Allplex™ STI Essential Assay but also HSV-2 (Table 4 and Table S3).

With respect to LoD verification, the experimentally observed results were consistent with the manufacturer-declared values (Table 5). It should be noted that this analysis was intended as a verification of LoD performance under local laboratory conditions, rather than a formal determination of the limit of detection. Triplicate testing was performed to confirm assay reproducibility at and around the declared LoD concentrations. As expected, lower target concentrations were associated with higher Ct values, and minor variations between replicates can be attributed to experiments conducted near the analytical detection limit. The LoD verification also allowed assessment of assay performance for HSV-1 and HSV-2, which were not included among routine clinical samples.

Overall, our results show that the performance of the STI STANDARD™ M10 panel kit in our clinical routine is superimposable to the test currently in use and that the LoD values indicated by the manufacturer have been verified.

## 5. Conclusions

The STANDARD™ M10 STI Panel demonstrated diagnostic performance comparable to that of the Allplex™ STI Essential Assay for the six shared targets evaluated, with overall sensitivity and specificity values ranging between 98% and 100% and an almost perfect level of agreement, as indicated by Cohen’s kappa analysis. Minor differences observed for *Neisseria gonorrhoeae*, reflected by a slightly lower sensitivity (89.5%) and negative predictive value (94.4%), are likely attributable to the limited number of positive samples and low target concentrations rather than to systematic analytical limitations of the assay. The verification of manufacturer-declared limits of detection under local laboratory conditions further supports the analytical reliability of the STANDARD™ M10 STI Panel. In addition, its short turnaround time (approximately 64 min), cartridge-based format and random-access capability represent relevant operational advantages that may facilitate integration into routine diagnostic workflows requiring rapid and on-demand testing. While ongoing and future studies on larger and more balanced sample sets will further strengthen the available evidence, particularly for non-urine specimen types, the STANDARD™ M10 STI Panel represents a valuable, reliable and user-friendly molecular tool for STI diagnosis and surveillance in clinical practice.

## Figures and Tables

**Table 1:** Reference materials and manufacturer-declared limits of detection (LoD) used for analytical sensitivity verification of the STANDARD™ M10 STI Panel [26].

Target	Declared LoD	Nominal Concentration *	Dilution Series	Supplier	Commercial Name	Reference Number
CT	3.8 IFU/mL	100,000; 10,000; 1000 copies/mL	4×, 2×, 1×, 0.5×, 0.25× LoD	SeraCare	AccuTrak™ CT/NG Verification Panel	2400-0245
NG	1.3 CFU/mL	100,000; 10,000; 1000 copies/mL				
TV	6 trophozoites/mL	100,000; 10,000; 1000 copies/mL	4×, 2×, 1×, 0.5×, 0.25× LoD	SeraCare	AccuTrak™ TV/MG Verification Panel	2400-0246
MG	0.02 bacteria/mL	100,000; 10,000; 1000 copies/mL				
HSV-1	7 TCID_50_/mL	10,000 copies/mL	4×, 2×, 1×, 0.5×, 0.25× LoD	ZeptoMetrix	NATtrol™ Herpes Simplex Virus Type 1	NATHSV1-0004
HSV-2	148 PFU/mL	10,000 copies/mL	4×, 2×, 1×, 0.5×, 0.25× LoD	ZeptoMetrix	NATtrol™ Herpes Simplex Virus Type 2	NATHSV2-0004
MH	0.06 CFU/mL	10,000–20,000 copies/µL (determined by qPCR)	4×, 2×, 1×, 0.5×, 0.25× LoD	Vircell	Amplirun^®^ Mycoplasma Hominis DNA control	MBC084
UU	0.5 CCU/mL	10,000–20,000 copies/µL (determined by qPCR)	4×, 2×, 1×, 0.5×, 0.25× LoD	Vircell	Amplirun^®^ Ureaplasma Urealyticum DNA control	MBC112

Notes: Abbreviations: CT, *Chlamydia trachomatis*; NG, *Neisseria gonorrhoeae*; TV, *Trichomonas vaginalis*; MG, *Mycoplasma genitalium*; MH, *Mycoplasma hominis*; UU, *Ureaplasma urealyticum*. IFU, inclusion-forming unit; CFU, colony-forming unit; CCU, color-changing unit; TCID_50_, 50% tissue culture infectious dose; PFU, plaque-forming unit. Each reference material was serially diluted in 0.9% sodium chloride solution to generate five testing levels (4×, 2×, 1×, 0.5× and 0.25× the declared LoD), which were tested for analytical sensitivity verification. * The concentrations listed in the “Nominal concentration” column correspond to the manufacturer-provided values of the quantified commercial standards used as source materials for LoD verification.

**Table 2:** Distribution of specimen types and number of positive samples included in the study.

Specimen Type	Total Tested (N)	Positive Samples (Any Target)	% Positive
Vaginal swabs (VSs)	113	61	54.0%
Rectal swabs (RSs)	18	9	56.0%
Urethral swabs (USs)	9	5	55.6%
Oropharyngeal swabs (OPSs)	7	2	28.6%
Seminal fluids (SFs)	3	1	33.3%
Total	150	78	52.0%

Notes: VS, vaginal swab; RS, rectal swab; US, urethral swab; OPS, oropharyngeal swab; SF, seminal fluid. Positive samples indicate specimens testing positive for at least one sexually transmitted infection target by either assay. Percentages are calculated relative to the total number of specimens tested for each specimen type.

**Table 3:** Samples positive for *Ureaplasma parvum* (UP) and corresponding cycle threshold (Ct) values detected by the Allplex™ STI Essential Assay.

Sample Number	UP (Ct)
54	POS (22.38)
56	POS (19.21)
57	POS (26.71)
58	POS (24.74)
61	POS (28.18)
62	POS (28.95)
63	POS (27.35)
64	POS (22.83)
70	POS (26.41)
72	POS (21.44)
74	POS (19.35)
84	POS (25.21)
85	POS (22.73)
88	POS (25.09)
89	POS (25.35)
90	POS (19.50)
92	POS (20.52)
94	POS (21.82)
95	POS (26.25)
96	POS (39.30)
101	POS (18.51)
131	POS (23.50)
132	POS (20.27)

Notes: UP, *Ureaplasma parvum*; Ct, cycle threshold. POS indicates a positive result obtained with the Allplex™ STI Essential Assay. *Ureaplasma parvum* is not included among the targets of the STANDARD™ M10 STI Panel and is reported here to evaluate potential analytical interference. Ct values are reported as provided by the comparator assay.

**Table 4:** Diagnostic performance of the STANDARD™ M10 STI Panel compared with the Allplex™ STI Essential Assay for each target organism.

Target	Sensitivity % [*n*/*N*; 95% CI]	Specificity % [*n*/*N*; 95% CI]	PPV	NPV	Accuracy [*n*/*N*; 95% CI]	κ (95% CI)
CT	95.65 [22/23; 78.1–99.9]	100.00 [127/127; 95.3–100]	100.00%	99.22%	99.3 [149/150; 96.3–100]	0.97 (0.92–1.00)
NG	89.47 [17/19; 65.5–98.2]	99.24 [130/131; 93.8–99.9]	98.48%	94.44%	98.0 [147/150; 94.2–99.5]	0.91 (0.79–1.00)
TV	100.00 [6/6; 54.1–100]	100.00 [144/144; 97.5–100]	100.00%	100.00%	100 [150/150; 97.6–100]	1.00 (1.00–1.00)
MG	92.86 [13/14; 66.1–99.8]	99.26 [135/136; 95.0–100]	92.86%	99.26%	98.7 [148/150; 95.1–99.9]	0.92 (0.82–1.00)
MH	100.00 [39/39; 91.0–100]	98.20 [109/111; 90.5–99.9]	95.12%	100.00%	98.7 [148/150; 95.0–99.9]	0.97 (0.90–1.00)
UU	97.30 [36/37; 86.1–99.9]	98.23 [111/113; 90.6–99.9]	94.74%	99.11%	98.0 [147/150; 94.3–99.5]	0.95 (0.88–1.00)

Notes: Sensitivity, specificity, positive predictive value (PPV), negative predictive value (NPV), overall accuracy and Cohen’s kappa (κ) are reported for each target organism, using the Allplex™ STI Essential Assay as the reference method. Numerators and denominators (*n*/*N*) indicate the number of concordant results over the total number of expected positive or negative samples. Ninety-five percent confidence intervals (95% CI) are shown in brackets. Confidence intervals for proportions were calculated using the Wilson score method, while κ confidence intervals were calculated using the asymptotic (Fleiss) method. Percentages are reported using the decimal separator applied throughout the manuscript.

**Table 5:** Discrepant results observed between the Allplex™ STI Essential Assay and the STANDARD™ M10 STI Panel.

Sample Number	Biological Matrix	Target Organism	Allplex™ STI Essential Assay (Ct)	STANDARD™ M10 STI Panel (Ct)
55	RS	NG	POS (32.98)	-
98	RS	UU, CT	POS (36.62) POS (36.11)	-
138	OPS	NG	POS (35.37)	-
140	RS	MG	POS (35.68)	-
75	VS	UU, MG	-	POS (34.38), POS (30.52)
104	OPS	NG	-	POS (25.32)
117	VS	MH	-	POS (30.60)
120	US	MH	-	POS (32.83)
149	RS	UU	-	POS (21.25)

Notes: Discrepant results are defined as samples yielding a positive result with only one of the two assays. Ct values are reported only for positive results; “-” indicates a negative result. For samples positive for more than one target organism, Ct values are listed in the same order as the corresponding targets. VS, vaginal swab; RS, rectal swab; US, urethral swab; OPS, oropharyngeal swab.

**Table 6:** Verification of the analytical limit of detection (LoD) for each target organism using quantified reference standards.

Target Organism	Declared LoD	Dilution	Equivalent Conc.	Replicate 1	Replicate 2	Replicate 3
CT	3.8 IFU/mL	4×	15.2	POS (31.37)	POS (32.84)	POS (34.15)
		2×	7.6	POS (31.91)	POS (35.01)	POS (34.40)
		1×	3.8	POS (34.49)	NEG	NEG
		0.5×	1.9	NEG	NEG	NEG
		0.25×	0.95	NEG	NEG	NEG
NG	1.3 CFU/mL	4×	5.2	POS (34.63)	POS (34.72)	POS (34.72)
		2×	2.6	NEG	POS (35.11)	POS (34.78)
		1×	1.3	POS (35.31)	NEG	POS (35.34)
		0.5×	0.65	NEG	NEG	NEG
		0.25×	0.33	NEG	NEG	NEG
TV	6 trophozoites/mL	4×	24	POS (32.49)	POS (31.99)	POS (31.94)
		2×	12	POS (34.89)	POS (35.34)	POS (32.92)
		1×	6	NEG	NEG	POS (34.88)
		0.5×	3	NEG	NEG	NEG
		0.25×	1.5	NEG	NEG	NEG
MG	0.02 bacteria/mL	4×	0.08	POS (31.00)	POS (31.94)	POS (31.92)
		2×	0.04	POS (32.31)	POS (33.71)	POS (33.90)
		1×	0.02	POS (34.85)	NEG	POS (32.22)
		0.5×	0.01	POS (35.34)	NEG	NEG
		0.25×	0.005	NEG	NEG	NEG
HSV-1	7 TCID_50_/mL	4×	28	POS (32.41)	POS (32.02)	POS (32.98)
		2×	14	POS (31.64)	POS (32.64)	POS (30.25)
		1×	7	POS (35.12)	POS (34.46)	POS (33.64)
		0.5×	3.5	NEG	NEG	NEG
		0.25×	1.75	NEG	NEG	NEG
HSV-2	148 PFU/mL	4×	592	POS (29.45)	POS (29.61)	POS (29.75)
		2×	296	POS (30.19)	POS (30.21)	POS (30.21)
		1×	148	POS (30.96)	POS (30.65)	POS (30.74)
		0.5×	74	NEG	POS (33.28)	NEG
		0.25×	37	NEG	NEG	NEG
MH	0.06 CFU/mL	4×	0.24	POS (31.04)	POS (30.76)	POS (31.92)
		2×	0.12	POS (32.18)	POS (33.48)	POS (32.41)
		1×	0.06	NEG	POS (34.25)	NEG
		0.5×	0.03	NEG	NEG	NEG
		0.25×	0.015	NEG	NEG	NEG
UU	0.5 CCU/mL	4×	2	POS (30.05)	POS (30.44)	POS (30.13)
		2×	1	POS (33.49)	POS (33.29)	POS (33.30
		1×	0.5	POS (34.74)	NEG	NEG
		0.5×	0.25	NEG	NEG	NEG
		0.25×	0.125	NEG	NEG	NEG

Notes: For each target organism, LoD verification was performed at five concentration levels corresponding to 4×, 2×, 1×, 0.5×, and 0.25× of the manufacturer-declared LoD, expressed in units per millilitre (units/mL). Each dilution level was tested in triplicate. “POS” indicates a positive amplification result, with Ct values reported in parentheses; “NEG” indicates no amplification detected. Declared LoD values correspond to those provided by the manufacturer for each target organism [26].

## Data Availability

The original contributions presented in this study are included in the article/Supplementary Material. Further inquiries can be directed to the corresponding author.

## Supplementary Materials

The following supporting information can be downloaded at https://www.mdpi.com/article/10.3390/microorganisms14030631/s1. Table S1. Results obtained by STANDARD M10 STI panel analyzing 50 samples previously identified as negative by Allplex STI Essential. Table S2. Results obtained by STANDARD M10 STI panel analyzing 100 samples previously identified as negative by Allplex STI Essential. Table S3. Sample detected positive by STANDARD M10 STI panel for HSV-1 or HSV-2.

## Abbreviations

The following abbreviations are used in this manuscript:

CFU	Colony-forming units
CI	Confidence interval
CT	*Chlamydia trachomatis*
Ct	Cycle threshold
ECDC	European Centre for Disease Prevention and Control
HICs	High-income countries
HSV-1	Herpes simplex virus type 1
HSV-2	Herpes simplex virus type 2
IFU	Instruction for use
κ (kappa)	Cohen’s kappa coefficient
LMICs	Low- and middle-income countries
LoD	Limit of detection
MG	*Mycoplasma genitalium*
MH	*Mycoplasma hominis*
NAATs	Nucleic acid amplification tests
NG	*Neisseria gonorrhoeae*
OPS	Oropharyngeal swabs
PCR	Polymerase chain reaction
PFU	Plaque-forming units
PID	Pelvic inflammatory disease
PoCt	Point of care testing
PPV	Positive predictive value
RS	Rectal swabs
SDGs	Sustainable Development Goals
SF	Seminal fluids
STI(s)	Sexually transmitted infection(s)
TCID_50_	50% tissue culture infectious dose
TV	*Trichomonas vaginalis*
UP	*Ureaplasma parvum*
US	Urethral swabs
UTM	Universal transport medium
UU	*Ureaplasma urealyticum*
VS	Vaginal swabs
WHO	World Health Organization
