# Supplementary material for: Evaluation of the Rapid and Cartridge-Based STANDARD™ M10 STI Panel: Analytical and Clinical Performance for Multiplex STI Detection
Source: Microorganisms. 2026 Mar 11;14(3):631. doi: 10.3390/microorganisms14030631 (PMC13029768; doi:10.3390/microorganisms14030631)
Supplement: Supplementary file 1 [file microorganisms-14-00631-s001.zip › microorganisms-4085069-supplementary.pdf]

Table S1. Results obtained by STANDARD M10 STI panel analyzing 50 samples previously identified as negative by Allplex STI Essential. NEG stands for negative sample for the specific target. CT = *Chlamydia trachomatis*; NG = *Neisseria gonorrhoeae*; MG = *Mycoplasma genitalium*, MH = *Mycoplasma hominis*, UU = *Ureaplasma urealyticum* and TV = *Trichomonas vaginalis*. VS = Vaginal Swab.

		Allplex™ STI Essential Assay						STANDARD™ M10 STI panel					
**Sample number**	**Biological matrix**	**CT**	**NG**	**MG**	**MH**	**UU**	**TV**	**CT**	**NG**	**MG**	**MH**	**UU**	**TV**
1	VS	NEG	NEG	NEG	NEG	NEG	NEG	NEG	NEG	NEG	NEG	NEG	NEG
2	VS	NEG	NEG	NEG	NEG	NEG	NEG	NEG	NEG	NEG	NEG	NEG	NEG
3	VS	NEG	NEG	NEG	NEG	NEG	NEG	NEG	NEG	NEG	NEG	NEG	NEG
4	VS	NEG	NEG	NEG	NEG	NEG	NEG	NEG	NEG	NEG	NEG	NEG	NEG
5	VS	NEG	NEG	NEG	NEG	NEG	NEG	NEG	NEG	NEG	NEG	NEG	NEG
6	VS	NEG	NEG	NEG	NEG	NEG	NEG	NEG	NEG	NEG	NEG	NEG	NEG
7	VS	NEG	NEG	NEG	NEG	NEG	NEG	NEG	NEG	NEG	NEG	NEG	NEG
8	VS	NEG	NEG	NEG	NEG	NEG	NEG	NEG	NEG	NEG	NEG	NEG	NEG
9	VS	NEG	NEG	NEG	NEG	NEG	NEG	NEG	NEG	NEG	NEG	NEG	NEG
10	VS	NEG	NEG	NEG	NEG	NEG	NEG	NEG	NEG	NEG	NEG	NEG	NEG
11	VS	NEG	NEG	NEG	NEG	NEG	NEG	NEG	NEG	NEG	NEG	NEG	NEG
12	VS	NEG	NEG	NEG	NEG	NEG	NEG	NEG	NEG	NEG	NEG	NEG	NEG
13	VS	NEG	NEG	NEG	NEG	NEG	NEG	NEG	NEG	NEG	NEG	NEG	NEG
14	VS	NEG	NEG	NEG	NEG	NEG	NEG	NEG	NEG	NEG	NEG	NEG	NEG
15	VS	NEG	NEG	NEG	NEG	NEG	NEG	NEG	NEG	NEG	NEG	NEG	NEG
16	VS	NEG	NEG	NEG	NEG	NEG	NEG	NEG	NEG	NEG	NEG	NEG	NEG
17	VS	NEG	NEG	NEG	NEG	NEG	NEG	NEG	NEG	NEG	NEG	NEG	NEG
18	VS	NEG	NEG	NEG	NEG	NEG	NEG	NEG	NEG	NEG	NEG	NEG	NEG
19	VS	NEG	NEG	NEG	NEG	NEG	NEG	NEG	NEG	NEG	NEG	NEG	NEG
20	VS	NEG	NEG	NEG	NEG	NEG	NEG	NEG	NEG	NEG	NEG	NEG	NEG
21	VS	NEG	NEG	NEG	NEG	NEG	NEG	NEG	NEG	NEG	NEG	NEG	NEG
22	VS	NEG	NEG	NEG	NEG	NEG	NEG	NEG	NEG	NEG	NEG	NEG	NEG
23	VS	NEG	NEG	NEG	NEG	NEG	NEG	NEG	NEG	NEG	NEG	NEG	NEG
24	VS	NEG	NEG	NEG	NEG	NEG	NEG	NEG	NEG	NEG	NEG	NEG	NEG
25	VS	NEG	NEG	NEG	NEG	NEG	NEG	NEG	NEG	NEG	NEG	NEG	NEG
26	VS	NEG	NEG	NEG	NEG	NEG	NEG	NEG	NEG	NEG	NEG	NEG	NEG
27	VS	NEG	NEG	NEG	NEG	NEG	NEG	NEG	NEG	NEG	NEG	NEG	NEG
28	VS	NEG	NEG	NEG	NEG	NEG	NEG	NEG	NEG	NEG	NEG	NEG	NEG
29	VS	NEG	NEG	NEG	NEG	NEG	NEG	NEG	NEG	NEG	NEG	NEG	NEG
30	VS	NEG	NEG	NEG	NEG	NEG	NEG	NEG	NEG	NEG	NEG	NEG	NEG
31	VS	NEG	NEG	NEG	NEG	NEG	NEG	NEG	NEG	NEG	NEG	NEG	NEG
32	VS	NEG	NEG	NEG	NEG	NEG	NEG	NEG	NEG	NEG	NEG	NEG	NEG
33	VS	NEG	NEG	NEG	NEG	NEG	NEG	NEG	NEG	NEG	NEG	NEG	NEG
34	VS	NEG	NEG	NEG	NEG	NEG	NEG	NEG	NEG	NEG	NEG	NEG	NEG
35	VS	NEG	NEG	NEG	NEG	NEG	NEG	NEG	NEG	NEG	NEG	NEG	NEG
36	VS	NEG	NEG	NEG	NEG	NEG	NEG	NEG	NEG	NEG	NEG	NEG	NEG
37	VS	NEG	NEG	NEG	NEG	NEG	NEG	NEG	NEG	NEG	NEG	NEG	NEG
38	VS	NEG	NEG	NEG	NEG	NEG	NEG	NEG	NEG	NEG	NEG	NEG	NEG

39	VS	NEG	NEG	NEG	NEG	NEG	NEG	NEG	NEG	NEG	NEG	NEG	NEG
40	VS	NEG	NEG	NEG	NEG	NEG	NEG	NEG	NEG	NEG	NEG	NEG	NEG
41	VS	NEG	NEG	NEG	NEG	NEG	NEG	NEG	NEG	NEG	NEG	NEG	NEG
42	VS	NEG	NEG	NEG	NEG	NEG	NEG	NEG	NEG	NEG	NEG	NEG	NEG
43	VS	NEG	NEG	NEG	NEG	NEG	NEG	NEG	NEG	NEG	NEG	NEG	NEG
44	VS	NEG	NEG	NEG	NEG	NEG	NEG	NEG	NEG	NEG	NEG	NEG	NEG
45	VS	NEG	NEG	NEG	NEG	NEG	NEG	NEG	NEG	NEG	NEG	NEG	NEG
46	VS	NEG	NEG	NEG	NEG	NEG	NEG	NEG	NEG	NEG	NEG	NEG	NEG
47	VS	NEG	NEG	NEG	NEG	NEG	NEG	NEG	NEG	NEG	NEG	NEG	NEG
48	VS	NEG	NEG	NEG	NEG	NEG	NEG	NEG	NEG	NEG	NEG	NEG	NEG
49	VS	NEG	NEG	NEG	NEG	NEG	NEG	NEG	NEG	NEG	NEG	NEG	NEG
50	VS	NEG	NEG	NEG	NEG	NEG	NEG	NEG	NEG	NEG	NEG	NEG	NEG

Table S2. Results obtained by STANDARD M10 STI panel analyzing 100 samples previously identified as negative by Allplex STI Essential. NEG and POS stands respectively for negative and positive sample for the specific target. When the sample is positive, the ct value identified by the test for the individual target is indicated. CT = *Chlamydia trachomatis*; NG = *Neisseria gonorrhoeae*; MG = *Mycoplasma genitalium*, MH = *Mycoplasma hominis*, UU = *Ureaplasma urealyticum* and TV = *Trichomonas vaginalis*. VS = Vaginal Swab, RS = Rectal Swab, OPS = Oropharyngeal Swab, US = Urethral swab, SF = Seminal fluids.

		**Allplex™ STI Essential Assay**						**STANDARD™ M10 STI panel**					
**Sample number**	**Biological matrix**	**CT**	**NG**	**MG**	**MH**	**UU**	**TV**	**CT**	**NG**	**MG**	**MH**	**UU**	**TV**
51	RS	NEG	NEG	NEG	POS (26,96)	POS (29,64)	NEG	NEG	NEG	NEG	POS (24,41)	POS (27,1)	NEG
52	VS	NEG	POS (19,83)	NEG	NEG	NEG	NEG	NEG	POS (22,2)	NEG	NEG	NEG	NEG
53	VS	POS (22,79)	NEG	NEG	NEG	NEG	NEG	POS (25,6)	NEG	NEG	NEG	NEG	NEG
54	VS	POS (22,46)	NEG	NEG	POS (27,9)	NEG	NEG	POS (22,52)	NEG	NEG	POS (24,61)	NEG	NEG
55	RS	NEG	POS (32,98)	NEG	NEG	POS (28,23)	NEG	NEG	NEG	NEG	NEG	POS (27,27)	NEG
56	VS	NEG	NEG	NEG	POS (21,1)	NEG	NEG	NEG	NEG	NEG	POS (20,12)	NEG	NEG
57	VS	NEG	NEG	NEG	POS (21,55)	POS (24,37)	NEG	NEG	NEG	NEG	POS (19,24)	POS (25,27)	NEG
58	VS	NEG	NEG	NEG	POS (23,09)	NEG	NEG	NEG	NEG	NEG	POS (23,85)	NEG	NEG
59	VS	NEG	POS (15,93)	NEG	NEG	NEG	NEG	NEG	POS (18,91)	NEG	NEG	NEG	NEG
60	RS	NEG	NEG	POS (27,5)	NEG	NEG	NEG	NEG	NEG	POS (31,61)	NEG	NEG	NEG
61	RS	NEG	NEG	POS (35,96)	NEG	POS (28,18)	NEG	NEG	NEG	POS (32,34)	NEG	POS (27,44)	NEG
62	RS	POS (29,41)	NEG	NEG	POS (19,63)	POS (28,95)	NEG	POS (32,1)	NEG	NEG	POS (19,4)	POS (28,06)	NEG
63	VS	NEG	NEG	NEG	POS (30,65)	NEG	NEG	NEG	NEG	NEG	POS (30,07)	NEG	NEG
64	VS	NEG	NEG	NEG	POS (15,69)	NEG	NEG	NEG	NEG	NEG	POS (15,29)	NEG	NEG
65	VS	NEG	NEG	NEG	POS (32,38)	NEG	NEG	NEG	NEG	NEG	POS (32,02)	NEG	NEG
66	RS	NEG	POS (24,1)	NEG	NEG	POS (32,55)	NEG	NEG	POS (35,02)	NEG	NEG	POS (27,61)	NEG
67	OPS	NEG	NEG	NEG	POS (34,82)	NEG	NEG	NEG	NEG	NEG	POS (34,84)	NEG	NEG

68	RS	NEG	POS (26,85)	NEG	NEG	POS (35,5)	NEG	NEG	POS (27,11)	NEG	NEG	POS (29,83)	NEG
69	RS	NEG	NEG	NEG	POS (21,2)	POS (30,24)	NEG	NEG	NEG	NEG	POS (20,77)	POS (29,2)	NEG
70	RS	NEG	NEG	NEG	POS (20,06)	NEG	NEG	NEG	NEG	NEG	POS (25,22)	NEG	NEG
71	VS	NEG	NEG	NEG	POS (16,48)	POS (20,08)	NEG	NEG	NEG	NEG	POS (15,78)	POS (19,4)	NEG
72	VS	NEG	NEG	NEG	POS (15,82)	NEG	NEG	NEG	NEG	NEG	POS (14,43)	NEG	NEG
73	VS	POS (24,59)	NEG	NEG	NEG	NEG	NEG	POS (29,17)	NEG	NEG	NEG	NEG	NEG
74	VS	NEG	NEG	NEG	POS (18,25)	NEG	NEG	NEG	NEG	NEG	POS (17,49)	NEG	NEG
75	VS	POS (14,83)	NEG	NEG	NEG	NEG	NEG	POS (13,2)	NEG	POS (30,52)	NEG	POS (34,38)	NEG
76	VS	NEG	NEG	POS (30,35)	NEG	NEG	NEG	NEG	NEG	POS (27,67)	NEG	NEG	NEG
77	OPS	NEG	NEG	NEG	POS (26,38)	NEG	NEG	NEG	NEG	NEG	POS (28,02)	NEG	NEG
78	VS	NEG	NEG	NEG	POS (22,69)	POS (18,87)	NEG	NEG	NEG	NEG	POS (21,65)	POS (18,97)	NEG
79	VS	NEG	NEG	NEG	NEG	POS (25,41)	NEG	NEG	NEG	NEG	NEG	POS (24,79)	NEG
80	VS	NEG	NEG	NEG	POS (14,55)	NEG	NEG	NEG	NEG	NEG	POS (12,69)	NEG	NEG
81	US	POS (32,03)	NEG	NEG	NEG	POS (24,94)	NEG	POS (29,73)	NEG	NEG	NEG	POS (26,99)	NEG
82	VS	NEG	NEG	NEG	NEG	POS (28,8)	NEG	NEG	NEG	NEG	NEG	POS (26,33)	NEG
83	VS	NEG	NEG	POS (33,68)	NEG	NEG	NEG	NEG	NEG	POS (28,96)	NEG	NEG	NEG
84	VS	NEG	NEG	POS (30,36)	NEG	NEG	NEG	NEG	NEG	POS (28,76)	NEG	NEG	NEG
85	VS	NEG	NEG	NEG	NEG	POS (35,5)	NEG	NEG	NEG	NEG	NEG	POS (33)	NEG
86	US	NEG	NEG	NEG	NEG	POS (22,75)	NEG	NEG	NEG	NEG	NEG	POS (20,62)	NEG
87	VS	NEG	NEG	NEG	NEG	NEG	POS (24,23)	NEG	NEG	NEG	NEG	NEG	POS (28,14)
88	VS	NEG	NEG	NEG	NEG	POS (29,61)	NEG	NEG	NEG	NEG	NEG	POS (27,68)	NEG
89	VS	POS (21,55)	NEG	NEG	NEG	NEG	NEG	POS (25,33)	NEG	NEG	NEG	NEG	NEG
90	VS	NEG	POS (16,68)	NEG	POS (16,54)	NEG	NEG	NEG	POS (19,19)	NEG	POS (15,48)	NEG	NEG
91	VS	NEG	NEG	NEG	POS (19,49)	POS (25,59)	NEG	NEG	NEG	NEG	POS (20,71)	POS (25,16)	NEG
92	VS	NEG	NEG	NEG	POS (29,58)	NEG	NEG	NEG	NEG	NEG	POS (27,58)	NEG	NEG
93	VS	NEG	NEG	NEG	POS (21,14)	NEG	NEG	NEG	NEG	NEG	POS (20,73)	NEG	NEG
94	VS	NEG	NEG	NEG	POS (18,13)	NEG	NEG	NEG	NEG	NEG	POS (16)	NEG	NEG
95	VS	NEG	NEG	NEG	NEG	POS (23,16)	NEG	NEG	NEG	NEG	NEG	POS (21,84)	NEG
96	VS	NEG	NEG	POS (29,24)	POS (18,32)	NEG	NEG	NEG	NEG	POS (26,76)	POS (15,89)	NEG	NEG
97	VS	POS (16,93)	NEG	NEG	POS (20,17)	NEG	NEG	POS (17,44)	NEG	NEG	POS (19,87)	NEG	NEG
98	RS	POS (36,11)	POS (22,64)	NEG	NEG	POS (36,62)	NEG	NEG	POS (23,9)	NEG	NEG	NEG	NEG
99	VS	NEG	NEG	NEG	NEG	POS (31,35)	NEG	NEG	NEG	NEG	NEG	POS (27,07)	NEG
100	VS	NEG	NEG	NEG	POS (29,95)	NEG	NEG	NEG	NEG	NEG	POS (27,55)	NEG	NEG
101	VS	NEG	NEG	NEG	POS (13,85)	NEG	NEG	NEG	NEG	NEG	POS (12,49)	NEG	NEG

102	VS	NEG	NEG	NEG	NEG	POS (31,73)	NEG	NEG	NEG	NEG	NEG	POS (31,42)	NEG
103	VS	NEG	NEG	NEG	POS (27)	POS (24,43)	NEG	NEG	NEG	NEG	POS (24,89)	POS (22,33)	NEG
104	OPS	POS (22,37)	NEG	NEG	NEG	NEG	NEG	POS (22,51)	POS (25,32)	NEG	NEG	NEG	NEG
105	VS	NEG	NEG	NEG	NEG	POS (23,02)	NEG	NEG	NEG	NEG	NEG	POS (25,21)	NEG
106	VS	NEG	NEG	NEG	NEG	POS (19,77)	NEG	NEG	NEG	NEG	NEG	POS (20,82)	NEG
107	VS	NEG	NEG	NEG	POS (29,52)	NEG	NEG	NEG	NEG	NEG	POS (29,99)	NEG	NEG
108	VS	NEG	NEG	NEG	POS (17,84)	NEG	NEG	NEG	NEG	NEG	POS (16,22)	NEG	NEG
109	VS	NEG	NEG	NEG	POS (35,96)	POS (29,95)	NEG	NEG	NEG	NEG	POS (32,14)	POS (29,93)	NEG
110	VS	NEG	NEG	NEG	POS (19,92)	POS (26,67)	POS (22,05)	NEG	NEG	NEG	POS (15,31)	POS (24,95)	POS (20,64)
111	VS	NEG	NEG	NEG	NEG	POS (23,57)	NEG	NEG	NEG	NEG	NEG	POS (24,24)	NEG
112	VS	POS (25,11)	NEG	NEG	POS (26,67)	POS (28,99)	NEG	POS (22,76)	NEG	NEG	POS (23,99)	POS (24,79)	NEG
113	VS	NEG	NEG	NEG	POS (33,6)	POS (36,16)	NEG	NEG	NEG	NEG	POS (30,5)	POS (34,04)	NEG
114	VS	POS (26,35)	NEG	NEG	NEG	NEG	NEG	POS (26,89)	NEG	NEG	NEG	NEG	NEG
115	VS	NEG	NEG	NEG	NEG	POS (24,5)	NEG	NEG	NEG	NEG	NEG	POS (21,35)	NEG
116	VS	NEG	NEG	NEG	POS (17,1)	NEG	NEG	NEG	NEG	NEG	POS (18,38)	NEG	NEG
117	VS	NEG	NEG	NEG	NEG	POS (25,26)	NEG	NEG	NEG	NEG	POS (30,6)	POS (24,53)	NEG
118	VS	NEG	NEG	NEG	NEG	POS (26,08)	NEG	NEG	NEG	NEG	NEG	POS (25,18)	NEG
119	VS	NEG	NEG	NEG	POS (15,62)	POS (23,02)	POS (29,05)	NEG	NEG	NEG	POS (15,54)	POS (21,24)	POS (29,86)
120	US	NEG	POS (19,39)	NEG	NEG	NEG	NEG	NEG	POS (20,01)	NEG	POS (32,83)	NEG	NEG
121	VS	POS (27,19)	NEG	NEG	NEG	NEG	NEG	POS (26,84)	NEG	NEG	NEG	NEG	NEG
122	US	POS (21,43)	NEG	NEG	NEG	NEG	NEG	POS (20,38)	NEG	NEG	NEG	NEG	NEG
123	VS	POS (17,23)	NEG	NEG	NEG	POS (27,11)	NEG	POS (16,37)	NEG	NEG	NEG	POS (22,12)	NEG
124	US	NEG	POS (19,33)	NEG	NEG	NEG	NEG	NEG	POS (20,17)	NEG	NEG	NEG	NEG
125	RS	POS (19,36)	NEG	NEG	POS (29,52)	POS (32,83)	NEG	POS (20,24)	NEG	NEG	POS (30,43)	POS (25,93)	NEG
126	VS	POS (19,41)	NEG	NEG	NEG	NEG	NEG	POS (21,58)	NEG	NEG	NEG	NEG	NEG
127	RS	POS (30,25)	NEG	NEG	NEG	NEG	NEG	POS (31,53)	NEG	NEG	NEG	NEG	NEG
128	US	NEG	NEG	NEG	NEG	NEG	POS (20,45)	NEG	NEG	NEG	NEG	NEG	POS (20,57)
129	VS	NEG	NEG	NEG	NEG	NEG	POS (21,92)	NEG	NEG	NEG	NEG	NEG	POS (21,62)
130	SF	NEG	POS (14,21)	NEG	NEG	NEG	NEG	NEG	POS (20,15)	NEG	NEG	NEG	NEG
131	VS	NEG	NEG	POS (33,76)	NEG	NEG	NEG	NEG	NEG	POS (31,1)	NEG	NEG	NEG
132	VS	POS (22,63)	NEG	NEG	NEG	NEG	NEG	POS (23)	NEG	NEG	NEG	NEG	NEG
133	VS	NEG	NEG	NEG	NEG	NEG	16,15	NEG	NEG	NEG	NEG	NEG	19,27
134	SF	POS (32,56)	NEG	NEG	NEG	NEG	NEG	POS (28,36)	NEG	NEG	NEG	NEG	NEG
135	US	POS (29,42)	NEG	NEG	NEG	NEG	NEG	POS (28,39)	NEG	NEG	NEG	NEG	NEG
136	SF	NEG	NEG	POS	NEG	NEG	NEG	NEG	NEG	POS	NEG	NEG	NEG

				(27,04)						(24,04)			
137	RS	POS (29,79)	POS (24,85)	NEG	NEG	NEG	NEG	POS (28,54)	POS (25,45)	NEG	NEG	NEG	NEG
138	OPS	NEG	POS (35,37)	NEG	NEG	NEG	NEG	NEG	NEG	NEG	NEG	NEG	NEG
139	US	NEG	POS (16,26)	NEG	POS (22,71)	NEG	NEG	NEG	POS (16,21)	NEG	POS (18,7)	NEG	NEG
140	RS	NEG	NEG	POS (35,68)	POS (24,81)	POS (33,06)	NEG	NEG	NEG	NEG	POS (26,54)	POS (31,99)	NEG
141	RS	NEG	NEG	POS (33,45)	NEG	NEG	NEG	NEG	NEG	POS (31,45)	NEG	NEG	NEG
142	OPS	NEG	POS (29,7)	NEG	NEG	NEG	NEG	NEG	POS (29,34)	NEG	NEG	NEG	NEG
143	OPS	NEG	POS (21,42)	NEG	NEG	NEG	NEG	NEG	POS (23,77)	NEG	NEG	NEG	NEG
144	RS	NEG	POS (20,54)	POS (38,9)	NEG	NEG	NEG	NEG	POS (22,65)	POS (34,79)	NEG	NEG	NEG
145	OPS	NEG	POS (23,99)	NEG	NEG	NEG	NEG	NEG	POS (25,61)	NEG	NEG	NEG	NEG
146	RS	NEG	POS (23,17)	POS (31,56)	NEG	NEG	NEG	NEG	POS (25,99)	POS (28,66)	NEG	NEG	NEG
147	VS	NEG	NEG	POS (33,41)	NEG	NEG	NEG	NEG	NEG	POS (34,9)	NEG	NEG	NEG
148	US	NEG	POS (19,35)	NEG	NEG	NEG	NEG	NEG	POS (21,35)	NEG	NEG	NEG	NEG
149	RS	NEG	NEG	POS (31,23)	NEG	NEG	NEG	NEG	NEG	POS (27,48)	NEG	POS (21,25)	NEG
150	VS	POS (13,84)	NEG	NEG	NEG	POS (26,23)	NEG	POS (17,45)	NEG	NEG	NEG	POS (29,68)	NEG

Table S3. Sample detected positive by STANDARD M10 STI panel for HSV-1 or HSV-2

Sample number	HSV-1/2 (ct)
62	POS HSV-2 (27,32)
64	POS HSV-1 (29,96)
69	POS HSV-2 (29,94)
81	POS HSV-2 (25,67)
120	POS HSV-2 (18,18)
